# Trichuriasis in Human Patients from Côte d’Ivoire Caused by Novel Species *Trichuris incognita* with Low Sensitivity to Albendazole/Ivermectin Combination Treatment

**DOI:** 10.3201/eid3101.240995

**Published:** 2025-01

**Authors:** Abhinaya Venkatesan, Rebecca Chen, Max Bär, Pierre H.H. Schneeberger, Brenna Reimer, Eveline Hürlimann, Jean T. Coulibaly, Said M. Ali, Somphou Sayasone, John Soghigian, Jennifer Keiser, John Stuart Gilleard

**Affiliations:** University of Calgary, Calgary, Alberta, Canada (A. Venkatesan, R. Chen, B. Reimer, J. Soghigian, J.S. Gilleard); Swiss Tropical and Public Health Institute, Allschwil, Switzerland (M. Bär, P.H.H. Schneeberger, E. Hürlimann, J. Keiser); University of Basel, Basel, Switzerland (M. Bär, P.H.H. Schneeberger, E. Hürlimann, J. Keiser); Université Félix Hophouët-Boigny, Abidjan, Côte d’Ivoire (J.T. Coulibaly); Public Health Laboratory Ivo de Carneri, Chake Chake, Pemba Island, Tanzania (S.M. Ali); Lao Tropical and Public Health Institute, Vientiane, Laos (S. Sayasone)

**Keywords:** trichuriasis, *Trichuris incognita*, whipworm, helminthiasis, albendazole, ivermectin, feces, albendazole/ivermectin combination treatment, next-generation sequencing, fecal DNA metabarcoding, parasites, Laos, Tanzania, Côte d’Ivoire, zoonoses

## Abstract

Albendazole/ivermectin combination therapy is a promising alternative to benzimidazole monotherapy alone for *Trichuris trichiura* control. We used fecal DNA metabarcoding to genetically characterize *Trichuris* spp. populations in patient samples from Côte d’Ivoire showing lower (egg reduction rate <70%) albendazole/ivermectin sensitivity than those from Laos and Tanzania (egg reduction rates >98%). Internal transcribed spacer (ITS) 1 and ITS2 metabarcoding revealed the entire detected Côte d'Ivoire *Trichuris* population was phylogenetically distinct from *T. trichiura* found in Laos and Tanzania and was more closely related to *T. suis*. Mitochondrial genome sequencing of 8 adult *Trichuris* worms from Côte d’Ivoire confirmed their species-level differentiation. Sequences from human patients in Cameroon and Uganda and 3 captive nonhuman primates suggest this novel species, *T. incognita*, is distributed beyond Côte d'Ivoire and has zoonotic potential. Continued surveillance by using fecal DNA metabarcoding will be needed to determine *Trichuris* spp. geographic distribution and control strategies.

*Trichuris trichiura* is a soil-transmitted helminth infecting 465 million persons globally, primarily in middle and low-income countries (*1*). Infections are most prevalent in children; moderate to severe infections cause chronic dysentery, diarrhea, and stunted growth (*2*). Control of this parasitic infection is largely dependent on preventive chemotherapy in high-risk disease-endemic regions by administering albendazole and mebendazole annually or biannually (*3*,*4*). Benzimidazole efficacy against *T. trichiura* is generally low; a meta-analysis comparing 38 clinical trials reported *T. trichiura* egg reduction rates (ERRs) were ≈50% for albendazole and ≈66% for mebendazole, and cure rates were ≈30% for albendazole and ≈42% for mebendazole (*5*). However, albendazole/ivermectin combination therapy improved efficacy against *T. trichiura* (*6**–**11*). Consequently, the World Health Organization has added this drug combination to its Essential Medicines List for soil-transmitted helminths (*3*,*11*).

A double-blind, parallel-group, phase 3, randomized controlled clinical trial recently showed an expected high efficacy of albendazole/ivermectin combination therapy against *T. trichiura* in Laos (ERR 99%), and Pemba Island, Tanzania (ERR 98%). However, albendazole/ivermectin combination therapy showed an unexpectedly low efficacy in Cȏte d’Ivoire (ERR 70%) (*12*).

Community-scale genetic analysis of soil-transmitted helminths is challenging because harvesting large numbers of parasites from patients is labor intensive and can be logistically difficult, whether harvesting adult worms from expulsion studies or parasite eggs from feces. DNA metabarcoding is a technique that enables detection of multiple taxa or genotypes from a single environmental sample through high-throughput DNA sequencing; applied directly to fecal DNA, this method offers a powerful alternative approach for helminth analysis. However, eggs from worms belonging to the genus *Trichuris* are robust and difficult to disrupt within fecal matter; together with the presence of fecal PCR inhibitors, egg processing is a challenge for PCR and metabarcoding methods applied directly to fecal DNA (*13*). Multiple studies have aimed to improve the amplification of *Trichuris* DNA from human fecal samples by including a bead-beating step to mechanically disrupt the eggs (*14*–*17*). We used a DNA extraction protocol that combined multiple freeze–thaw cycles and mechanical disruption and applied metabarcoding to internal transcribed spacer (ITS) 1 and ITS2 rRNA gene regions to genetically characterize *Trichuris* populations in fecal samples. 

## Materials and Methods

### DNA Extraction

We examined fecal samples preserved in ethanol from patients enrolled in a previously reported clinical trial (*12*). We prepared DNA from pretreatment fecal samples collected from patients in Cȏte d’Ivoire (n = 22), Laos (n = 36), and Pemba Island, Tanzania (n = 29). *Trichuris* egg counts were 91–1,151 eggs per gram (EPG) of feces (Appendix Table 1). We washed ≈250 mg of each fecal sample (in 90% ethanol) with molecular-grade water 3 times (1:2 ratio of feces:water) and centrifuged at 12,000 × *g*. We then performed 3 cycles of snap freezing in liquid nitrogen and 15 minutes of heating at 100°C with shaking at 750 rpm followed by vigorous bead beating for 3 minutes by using a Mini-Beadbeater-96 (Thomas Scientific, https://www.thomassci.com). We extracted DNA by using the QIAamp PowerFecal Pro DNA Kit (QIAGEN, https://www.qiagen.com) (Appendix).

We picked whole worms directly from patients’ fecal samples after anthelmintic treatment during an expulsion study in Côte d’Ivoire (M.A. Bär et al., unpub. data, https://doi.org/10.1101/2024.06.11.598441). We washed worms with sterile phosphate-buffered saline and stored them in absolute ethanol. We extracted DNA from separated worm heads by using a DNeasy Blood and Tissue Kit (QIAGEN).

### Fecal DNA Metabarcoding

We designed PCR primers to amplify the following *Trichuris* genetic markers in fecal DNA: ITS1 (733-bp amplicon), ITS2 (592 bp), *cox-1* (430 bp), *nad-1* (470 bp), and *nad-4* (446 bp) (Appendix Table 2). Each PCR reaction comprised 5 μL of 5X KAPA HiFi buffer (Roche, https://www.roche.com), 0.75 μL of 10 μmol/L Illumina-adapted forward primer and 0.75 μL of 10 μmol/L Illumina-adapted reverse primer (Illumina, https://www.illumina.com), 0.75 μL of 10 μmol/L dNTPs (Roche), 0.5 μL KAPA HiFi Hotstart polymerase (0.5 units) (Roche), 0.1 μL bovine serum albumin (Thermo Fisher Scientific, https://www.thermofisher.com), 15.15 μL molecular-grade water, and 2 μL of extracted fecal DNA. Thermocycling conditions were 95°C for 3 minutes, followed by 40 cycles of 98°C for 20 seconds, 65°C (ITS-1), 64°C (ITS-2, *nad-1*, *nad-4*), 60°C (*cox-1)*, or 63°C (β-tubulin) for 15 seconds, and 72°C for 30 seconds; and then 72°C for 2 minutes. We prepared a pooled library from amplicons as previously described (M.A. Bär et al., unpub. data). We sequenced amplicons on an MiSeq instrument (Illumina) by using Illumina V3 (2 × 300 bp) sequencing chemistry for ITS2, *nad-1,* and *nad-4*, and V2 (2 × 250 bp) sequencing chemistry for ITS1 *and cox-1*. DNA amplicon sequencing data from this study have been deposited in the National Center for Biotechnology Information Sequence Read Archive (https://www.ncbi.nlm.nih.gov/sra; BioProject no. PRJNA1131306).

We performed quality filtering of paired-end reads and primer removal by using Cutadapt version 3.2 (*19*) and analyzed the adaptor-trimmed paired-end reads by using DADA2 as previously described (*20*,*21*). We trimmed forward reads to 240 bp for the ITS1, ITS2, *cox-1, nad-1*, and *nad-4* amplicons and trimmed reverse reads to 190 bp (ITS1), 220 bp (ITS2), 230 bp (*cox-1*), 220 bp (*nad-1*), and 220 bp (*nad-4*). We removed reads if they were <50 bp or had an expected error rate of >1 (ITS1), >3 (ITS2), >1 (*cox-1*), >2 (*nad-1*), and >2 (*nad-4*) nucleotides for forward reads and >2 (ITS1), >5 (ITS2), >2 (*cox-1*), >5 (*nad-1*), and >5 (*nad-4*) nucleotides for reverse reads. We aligned amplicon sequence variants (ASVs) to reference sequences (Appendix Table 3) by using the MAFFT tool for multiple sequence alignment (*22*), and we removed off-target ASVs. We performed manual correction of sequence alignments by using Geneious version 10.0.9 (Geneious, https://www.geneious.com). In the final analysis, we only included samples that had a total read depth of >1,000 mapped reads, >0.1% ASVs in the population overall, and >200 reads in an individual sample.

### Phylogenetic and Population Genetic Analysis of Metabarcoding Data

We performed multiple sequence alignments for *Trichuris* ITS1, ITS2, *cox-1*, *nad-1*, and *nad-4* reference sequences from GenBank (Appendix Table 3) and for the ASVs from amplicon sequencing by using the MAFFT tool as described. After manual correction for indels in Geneious software, we performed phylogenetic analysis by using the maximum-likelihood method. We constructed statistical parsimony haplotype networks from the corrected ASV alignments by using the pegas package (*23*) in R (The R Project for Statistical Computing, https://www.r-project.org). We then visualized and annotated those networks by using Cytoscape version 3.9.1 (*24*).

After ASV filtering and alignment, we generated haplotype distribution bar charts in R by using the ggplot2 package (*25*). We calculated the Shannon-Wiener index for α diversity by using the vegan package (https://github.com/vegandevs/vegan) in R and plotted each population by using ggplot2. We used pairwise *t*-tests to determine significant differences in the α diversity between populations. For β diversity analysis, we used a Bray-Curtis dissimilarity matrix to calculate differences in relative abundance and Jaccard distance to calculate the presence or absence of ASVs among the samples. We performed principal coordinate analysis by using the vegan package in R and generated those plots by using ggplot2. We calculated nucleotide diversity and ASV heterozygosity by using the pegas package, whereas we performed pairwise fixation index (F_ST_) calculations and significance testing by using Arlequin version 3.5 (*26*) and 1,000 permutations.

### Adult Worm Whole-Genome Sequencing and Analysis

We prepared adult worm sequencing libraries by using the NEBNext Ultra II FS DNA Library Prep Kit (New England Biolabs, https://www.neb.com) and measured DNA concentrations by using the Invitrogen dsDNA High Sensitivity Assay Kit and Qubit 4 Fluorometer (Thermo Fisher Scientific) and 2 μL of DNA extract as input. We checked fragment sizes by using 1% gel electrophoresis and sequence the libraries by using 2 × 150–bp paired-end chemistry on an Illumina MiSeq instrument.

After whole-genome sequencing, we used *fastp* (*27*) to filter reads below a Phred quality score of 15 and to trim adapters from paired-end reads. Next, we used GetOrganelle (*28*) to assemble mitochondrial genomes and extract ITS1 and ITS2 sequence data from each sample. For assembling mitochondrial genomes, we ran GetOrganelle with the flags “-R 10,” “-k 21, 45, 65, 85, 105” and a default animal mitochondrial genome database seed (-F animal_mt). We then annotated mitochondrial genomes in Geneious version 10.0.9 by using the “annotate from” function for *Trichuris* reference genomes from GenBank (Appendix Table 3) with a threshold of 90% similarity. For extracting ITS1 and ITS2 sequences, we used 2 *T. suis* sequences from GenBank (accessions nos. AM993005 and AM993010) as seeds; we used -F set to “anon” and a target size of 1,350 bp.

We downloaded additional mitochondrial genomes representing 13 described species of *Trichuris*, several undescribed *Trichuris* lineages, and *Trichinella* spp. outgroups from GenBank and sequences from a previously published study (Appendix Table 3) (*29*). We extracted protein coding sequences for the genes *atp-6, cox-1, cox-2, cox-3, cytb, nad-1, nad-2, nad-3, nad-4, nad-4L, nad-5,* and *nad-6* from all 45 downloaded mitochondrial genomes; we aligned each sequence separately by using MAFFT. We calculated pairwise nucleotide identity by using Geneious. We used IQ-TREE 2 (*30*) to estimate the maximum-likelihood phylogeny from the concatenated alignment of the 12 protein-coding genes and estimated a separate substitution model per gene (using flag -m ModelFinder Plus). We assessed support for branches in the maximum-likelihood topology by using both ultrafast bootstrap (1,000 replicates; -B 1000) and a Shimodaira–Hasegawa–like approximate likelihood ratio test (–alrt 1000). We visualized the resulting maximum-likelihood phylogeny by using FigTree (https://github.com/rambaut/figtree) and rooted the tree on the branch leading to *Trichinella* spp*.*

## Results

### ITS1 and ITS2 rDNA Metabarcoding of Côte d’Ivoire Samples

We amplified and sequenced a 733-bp ribosomal ITS1 region from 21 (Cȏte d’Ivoire), 34 (Laos), and 29 (Pemba Island) fecal samples and a 592-bp ITS2 region from 15 (Cȏte d’Ivoire), 26 (Laos), and 23 (Pemba Island) fecal samples. Paired-end reads were merged to produce a 461-bp sequence for ITS1 with an average mapped read depth of 17,246 (range 2,205–28,402) and a 457-bp sequence for ITS2 with an average read depth of 7,660 (range 1,172–18,027).

**Figure 1:**
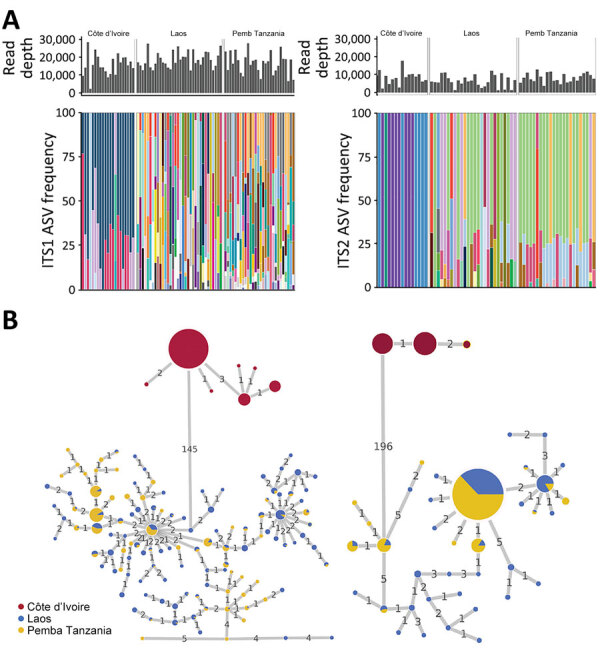
Frequencies and haplotype networks for ASVs of *Trichuris* spp. ITS1 and ITS2 in study of novel *Trichuris incognita* identified in patient fecal samples from Côte d’Ivoire and reference sequences. A) Histograms indicate the sequencing read depths and bar plots indicate the relative frequencies of ASVs generated by amplicon sequencing of *Trichuris* ITS1 (left) and ITS2 (right) loci from samples collected in Côte d’Ivoire, Laos, and Pemba Island, Tanzania. Colored bars indicate similarities or differences in ASV frequencies between the 3 geographic regions. B) Statistical parsimony haplotype networks of ASVs for *Trichuris* ITS1 and ITS2 loci generated by amplicon sequencing of fecal samples from patients in Côte d’Ivoire, Laos, and Pemba Island, Tanzania. Colored circles indicate the region and size of each circle indicates the ASV frequency. Numbers on connecting lines indicate the number of nucleotide differences between adjacent haplotypes. ASV, amplicon sequencing variant; ITS, internal transcribed spacer.

Many ASVs were shared between the *Trichuris* populations in Laos and Pemba Island, but no ASVs were shared with the Cȏte d’Ivoire population (Figure 1, panel A). Pairwise F_ST_ values were higher in Cȏte d’Ivoire when compared with either Laos and Pemba Island values than values between Laos and Pemba Island (Table). The ITS1 and ITS2 ASVs in the Cȏte d’Ivoire *Trichuris* population were genetically divergent from those found in the Laos and Pemba Island populations, separated by 145/461 nt difference for ITS1 and 196/457 nt differences for ITS2 (Figure 1, panel B).

**Table:** Pairwise F_ST_ comparisons among *Trichuris* populations from different regions in study of novel *T. incognita* identified in patient fecal samples from Côte d’Ivoire*

Region	ITS1 F_ST_			ITS2 F_ST_	
	Laos	Pemba		Laos	Pemba
Côte d’Ivoire	0.248	0.273		0.300	0.336
Laos	NA	0.017		NA	0.015

*Pairwise F_ST_ comparisons were made between *Trichuris* spp. populations found in patient fecal samples from Laos; Pemba Island, Tanzania; and Côte d’Ivoire. F_ST_, fixation index; ITS, internal transcribed spacer; NA, not applicable.

**Figure 2:**
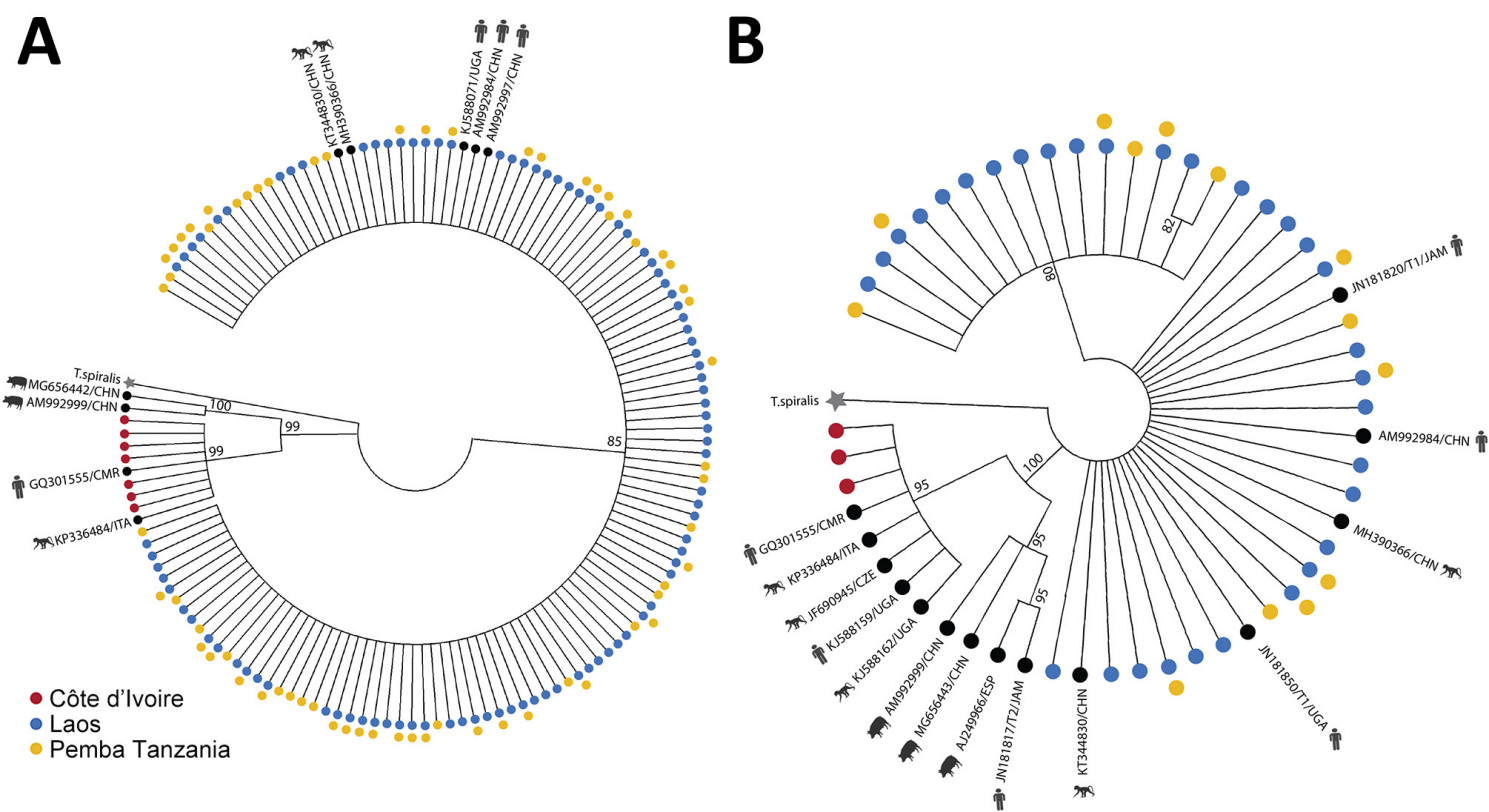
Phylogenetic analysis of ITS1 and ITS2 rRNA ASVs in study of novel *Trichuris incognita* identified in patient fecal samples from Côte d’Ivoire. Maximum-likelihood method was used to construct trees for *Trichuris* ITS1 (A) and ITS2 (B) ASVs from Côte d’Ivoire, Laos, and Pemba Island, Tanzania, as well as additional *Trichuris* reference sequences from pigs, humans, and nonhuman primates in GenBank. Trees were generated by amplicon sequencing of fecal sample DNA from patients in Côte d’Ivoire, Laos, and Pemba Island, Tanzania. After generating the clusters, trees were transformed into cladograms for visualization. *Trichinella spiralis* (GenBank accession no. KC006432) was used as the outgroup. Each tip of the tree is an ASV or a sequence from GenBank. Colors indicate the 3 regions. Black circles indicate GenBank sequences. Kimura 80 model for ITS-1 and general time reversible model for ITS-1 were chosen as the best nucleotide substitution models. Models were chosen by using jModeltest version 2.1.10 (https://github.com/ddarriba/jmodeltest2). Trees were constructed by using PhyML v3.3 (https://github.com/stephaneguindon/phyml) with 100 bootstrap replicates. Trees were condensed by using MEGA version 11 (https://www.megasoftware.net) to only display branches with consensus support >80%. Trees not to scale. ASV, amplicon sequencing variant; ITS, internal transcribed spacer.

**Figure 3:**
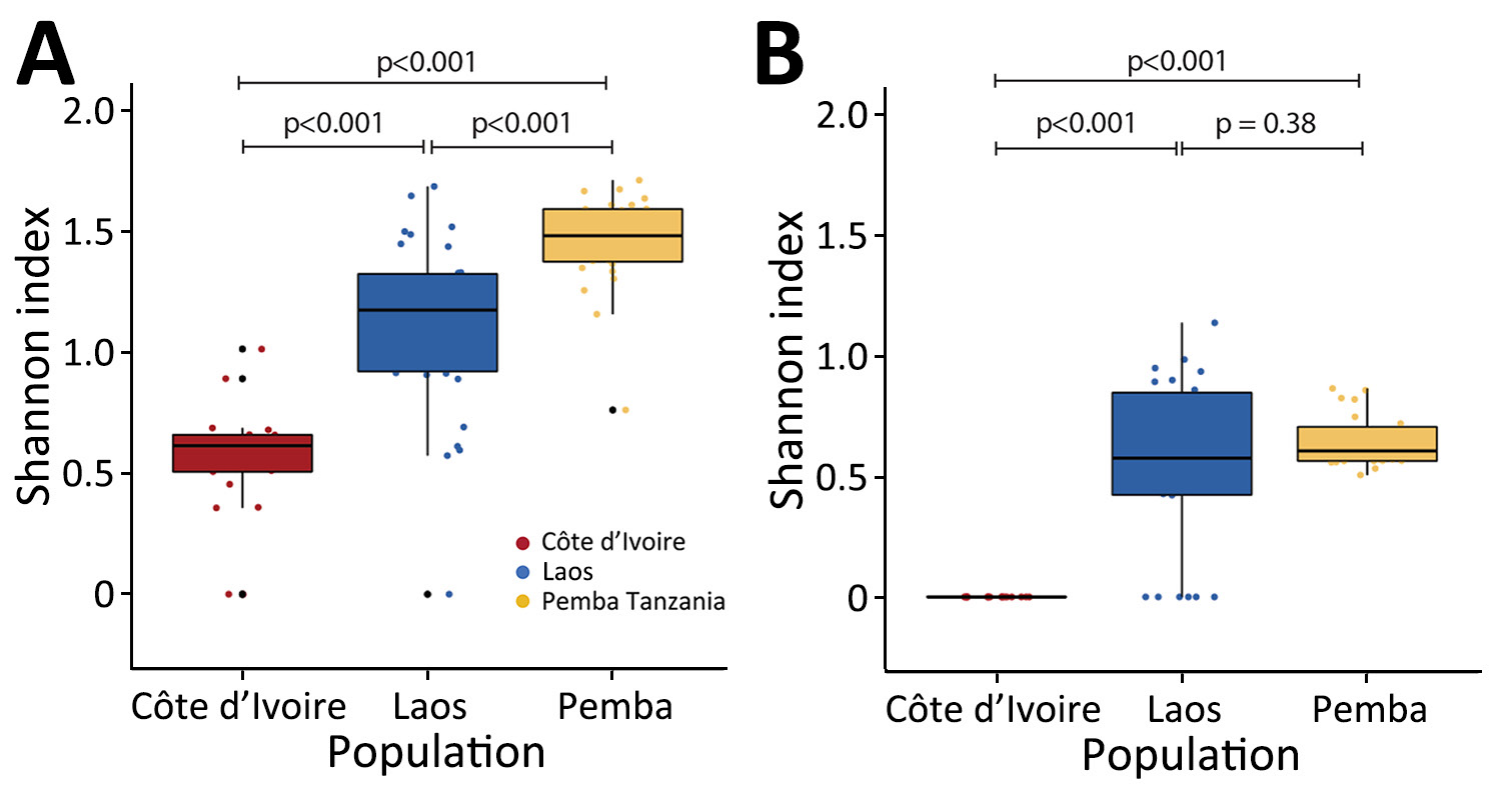
Alpha diversity of ITS1 and ITS2 rRNA amplicon sequence variants in study of novel *Trichuris incognita* identified in patient fecal samples from Côte d’Ivoire. Shannon-Wiener Index values, indicating α diversity, were determined for *Trichuris* spp. ITS1 (A) and ITS2 (B) amplicon sequence variants generated by sequencing fecal sample DNA from patients in Cote d’Ivoire, Laos, and Pemba Island, Tanzania. Horizontal lines within boxes indicate medians; box tops and bottoms indicate upper (third) and lower (first) quartiles; error bars (whiskers) indicate minimum and maximum values. Pairwise *t*-tests were used to determine p values. ITS, internal transcribed spacer.

Phylogenetic analysis of *Trichuris* ITS1 and ITS2 ASVs from the 3 geographic regions had broadly congruent results for those 2 markers (Figure 2). All *Trichuris* ASVs from Cȏte d’Ivoire formed a phylogenetic clade separate from those from Laos and Pemba Island, clustering with *Trichuris* reference sequences from human patients in Cameroon and Uganda and from captive nonhuman primates from Italy, Czech Republic, and Uganda. This clade was more closely related to the clade containing *T. suis* than that containing ASVs from Laos and Pemba Island for both markers (Figure 2). *Trichuris* ITS2 and ITS1 ASVs from Côte d’Ivoire shared 65% (ITS1) and 80% (ITS2) identity with *T. suis* sequences but only 44% (ITS-1) and 56% (ITS2) identity with ASVs from Laos and Pemba Island. ASV heterozygosity and nucleotide diversity for both ITS1 and ITS2 were higher in samples from Pemba Island and Laos with much higher α diversity than in samples from Cȏte d’Ivoire (Figure 3; Appendix Table 4).

### Mitochondrial Gene Metabarcoding and Population Substructuring

Species-specific primers designed by using *T. trichiura* reference sequences (GenBank accession nos. NC_017750, GU385218, AP017704, and KT449825) successfully generated mitochondrial *cox-1, nad-1*, and *nad-4* amplicons for metabarcoding of most fecal samples from Laos and Pemba Island but not for any samples from Cȏte d’Ivoire, suggesting primer site sequence polymorphism of those genes in the *Trichuris* population of Cȏte d’Ivoire (Appendix Figure 1). Mitochondrial *cox-1, nad-1,* and *nad-4* ASVs generated from Laos and Pemba Island fecal samples revealed high α diversity (Appendix Figures 2, 3), and all ASVs clustered phylogenetically with *T. trichiura* reference sequences, supporting their species identity (Appendix Figure 4). Although pairwise F_ST_ values between *T. trichiura* populations from Pemba Island and Laos were low (0.024 [*cox-1*], 0.002 [*nad-1*], and 0.142 [*nad-4*]), multidimensional metric β diversities and haplotype networks indicated some geographic substructuring between those regions, particularly for the *nad-1* gene marker (Appendix Figures 5, 6).

### Analysis of Complete Mitochondrial Genomes in Adult Worms from Côte d’Ivoire

**Figure 4:**
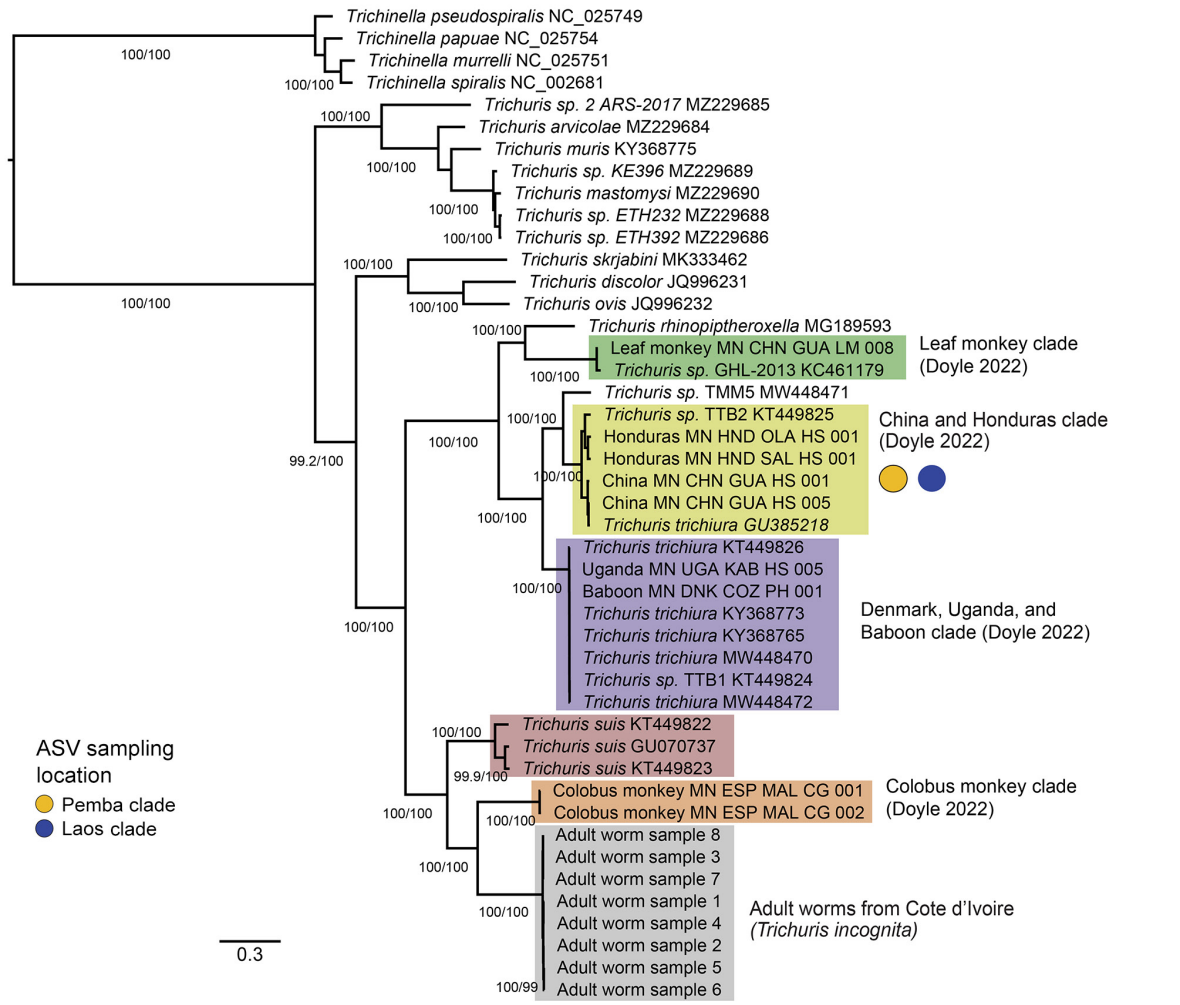
Phylogenetic analysis of *Trichuris* spp. from complete mitochondrial genome sequences in study of novel *Trichuris incognita* identified in patient fecal samples from Côte d’Ivoire. Tree was reconstructed by using the maximum-likelihood method for 12 mitochondrial protein coding genes from *Trichuris* spp. compared with sequences from GenBank. Tree was constructed by using IQ-TREE; alignments were made for the 12 protein coding genes from 45 mitochondrial genomes, including 8 *T. incognita* sequences obtained from an expulsion study of patients in Côte d’Ivoire (M.A. Bär et al., unpub. data, https://doi.org/10.1101/2024.06.11.598441). Ultrafast bootstrap/Shimodaira-Hasegawa–like branch support values >95/95 are indicated on branches. Color-shaded boxes indicate clades previously identified in the literature. Yellow (Pemba Island) and blue (Laos) colored circles indicate where the clades mapped according to *cox-1*, *nad-1,* and *nad-4* mitochondrial ASVs. Scale bar indicates nucleotide substitutions per site. ASV, amplicon sequencing variant.

The primers designed by using *T. trichiur*a mitochondrial reference sequences did not yield amplicons from the Cȏte d’Ivoire fecal DNA samples; therefore, we extracted and assembled complete mitochondrial genomes from whole-genome sequencing data obtained from 8 adult *Trichuris* worms collected in a separate anthelmintic expulsion study in Cȏte d’Ivoire (M.A. Bär et al., unpub. data). Average mitochondrial genome coverage was 2,541×, and the 8 assembled mitochondrial genomes ranged from 14,253 bp to 14,663 bp (average 14,338.13 bp), slightly larger than *T. trichiura* reference genomes retrieved from GenBank (range 14,046–14,091; n = 6) but similar to those of *T. suis* (range 14,436–14,521; n = 3). Phylogenetic reconstruction of protein coding genes from *Trichuris* mitochondria indicated that the 8 Cȏte d’Ivoire mitochondrial genomes formed a single, well-supported clade, sister to *Trichuris* from *Colobus* monkeys (*29*) and more genetically distant from *T. trichiura* (Figure 4). Despite this phylogenetic similarity, adult worms sampled from Cȏte d’Ivoire were genetically distinct from those collected from *Colobus* monkeys as well as *T. suis* worms; pairwise nucleotide identities of DNA from Cȏte d’Ivoire were 80.2% (compared with *Colobus* monkeys) and 78.9% (compared with *T. suis*) for *cox-1* and 73.1% (compared with *Colobus* monkey) and 71.7% (compared with *T. suis*) across all protein-coding genes (Appendix Table 5).

The ITS1 and ITS2 rDNA sequences recovered from the whole-genome sequences of the 8 adult worms were aligned to the ASVs generated from fecal metabarcoding. Sequence identities ranged from 93.5% to 99.8%, indicating that the same species of *Trichuris* was sampled in both the adult worm sequences and the fecal DNA metabarcoding (Appendix Figures 7, 8).

## Discussion

Co-administration of benzimidazoles with macrocyclic lactones provides much higher efficacy against *T. trichiura* than monotherapy with either drug class (*5*–*10*). A double-blind, randomized, controlled trial was previously conducted to compare the efficacy of an albendazole/ivermectin combination with albendazole monotherapy in Cȏte d’Ivoire, Laos, and Tanzania (*12*). Although the combination therapy had much higher efficacy than albendazole monotherapy in Laos and Tanzania, the efficacy in Cȏte d’Ivoire was lower and comparable to that observed for monotherapy. We used short-read metabarcoding of several taxonomic markers to compare the genetics of the *Trichuris* populations in fecal samples from multiple patients at each of the same 3 study sites. Analysis of the ITS1 and ITS2 ASVs revealed a genetically divergent *Trichuris* population in Cȏte d’Ivoire that did not share any ASVs with the samples in Laos or Pemba Island (Figure 1). Pairwise F_ST_ calculations and haplotype networks revealed substantial population differentiation in the Cȏte d’Ivoire *Trichuris* population.

The ASVs of both ITS1 and ITS2 rRNA DNA regions clustered into 2 distinct phylogenetic clades, clades A and B (Figure 2). All ASVs from patients in Laos and Pemba Island belonged to clade A, which corresponds to the most described reference sequences for *T. trichiura* in public databases (*31*–*38*). Various nomenclature designations have been used in public databases and the literature for *Trichuris* sequences within clade A: subclade DG (*32*), group 1 (*33*), clade DG (*36*), clade 2 (*37*), or subgroup 1 (*38*). In contrast, all *Trichuris* ASVs generated from Côte d’Ivoire patient samples fell into a second monophyletic clade B, which were phylogenetically closer to reference sequences from the porcine parasite *T. suis* than to the human parasite *T. trichiura* (Figure 2). Nevertheless, clade B is distinct from *T. suis* and is more closely related to a small number of reference sequences from humans and nonhuman primates previously deposited in public databases (*32*,*33*,*36*–*38*). Specifically, clade B sequences are related to 1 human patient from Cameroon, 1 human patient from Uganda (*36*), and several additional reference sequences from red *Colobus* monkeys in Uganda, captive vervet monkeys in Italy, and captive lion-tailed macaques in the Czech Republic (*32*,*33*) (Figure 2). The nomenclature previously used in the literature for this sequence group is also varied: subclade CA (*32*), group 3 (*33*), CP-GOB (*36*), clade 1 (*37*), or subgroup 5 (*38*).

Primers designed to amplify *T. trichiura* mitochondrial *cox-1*, *nad-1* and *nad-4* reference sequences consistently generated PCR amplicons from samples from Laos and Pemba Island, but not from Côte d’Ivoire. The PCR amplification failure for the Côte d’Ivoire samples is unsurprising given the genetic divergence of this *Trichuris* population from *T. trichiura*. Although some genetic substructuring in the *Trichuris* population was apparent in samples from Laos and Pemba Island according to mitochondrial DNA metabarcoding, all ASVs from those 2 sites clustered with human-derived *T. trichiura* reference sequences from public databases, including sequences from China, Japan, and Honduras, and with *Trichuris* sequences from captive baboons in the United States (*29*) (Appendix Figure 4). Together with the ITS1 and ITS2 ASV data, the ASV data indicate the albendazole/ivermectin-sensitive *Trichuris* populations from Laos and Pemba Islane are entirely composed of *T. trichiura*.

**Figure 5:**
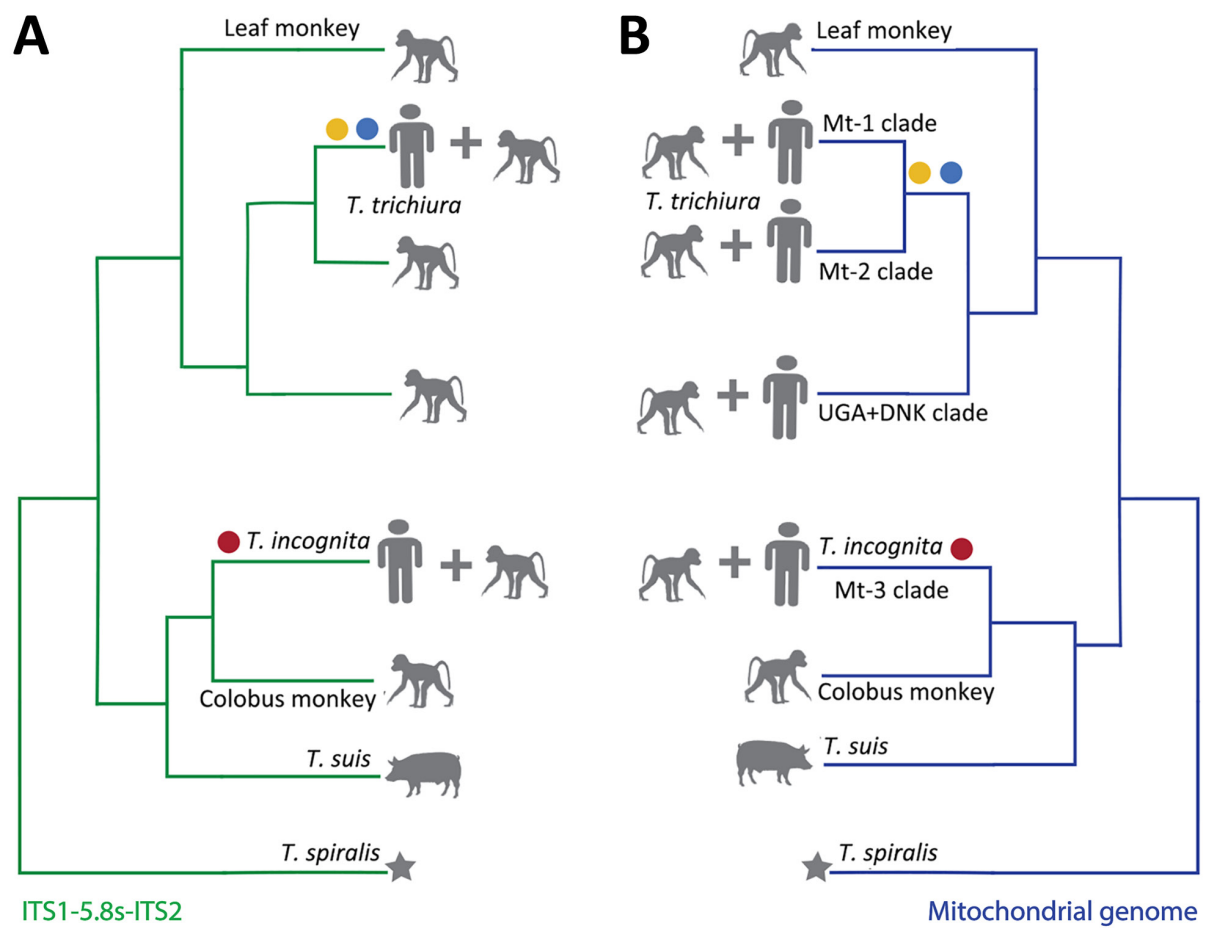
Schematic of phylogenetic relationships of *Trichuris* spp. infecting humans and nonhuman primates adapted from previously published studies. Relationships are indicated for the ribosomal ITS1-5.8S-ITS2 region (A) and the mitochondrial genome (B). Two major clades of *Trichuris* in the ribosomal DNA and mitochondrial DNA phylogenies infected both humans and nonhuman primates. Yellow circle indicates *T. trichiura* from Pemba Island, blue indicates *T. trichiura* from Laos, and red circle indicates *T. incognita* from Côte d’Ivoire. Pig-derived *T. suis* is also included in the tree as a reference. Star indicates *Trichinella spiralis*, used as an outgroup.

To investigate the mitochondrial phylogeny of the *Trichuris* sequences, we assembled the complete mitochondrial genomes from 8 adult *Trichuris* worms collected from an expulsion study conducted in Côte d’Ivoire (M.A. Bär et al., unpub. data). Although the mitochondrial genome sequences from Côte d’Ivoire clarify their phylogenetic relationship to other *Trichuris* spp. sequences, the lack of amplicon sequencing data limits our ability to assess population diversity. Phylogenetic analysis of the 8 assembled mitochondrial genomes, in conjunction with 37 previously released mitochondrial genomes (*29*), produced a similar outcome observed for the ITS marker analysis. Those 8 mitochondrial genomes are more closely related to *Trichuris* spp. from *Colobus* monkeys and *T. suis* than they are to *T. trichiura* (Figure 4). Despite the phylogenetic relationship of *Trichuris* mitochondrial genes from Côte d’Ivoire to those of *Trichuris* spp. from other animals, the genetic distances between lineages, shown in our phylogenetic analysis and pairwise nucleotide identity analysis (Appendix Table 5), indicate that we have sampled a new species of *Trichuris*. The reference genome of this *Trichuris* species, proposed to be *T. incognita*, has been characterized concurrently with this study (M.A. Bär, et al. unpub. data), and phylogenetic relationships were determined between *T. trichiura*, *T. suis*, and *T. incognita* (Figure 5).

The study site in Côte d’Ivoire has the longest record of consistent use of albendazole/ivermectin treatment in mass drug administration (MDA) campaigns aimed at combating lymphatic filariasis (>2 decades) (*12*,*39*). This record contrasts with that in Pemba Island, where the lymphatic filariasis MDA ended many years ago, and that in Laos, where ivermectin has not been previously used (*12*). It is possible that this treatment history has provided a selective advantage for *T. incognita* in Côte d’Ivoire because of an inherently lower sensitivity to this drug combination compared with *T. trichiura*.

Despite the higher (mean EPG 350 [range 110–1,151]) fecal egg counts in fecal samples from Côte d’Ivoire than those in Laos (mean EPG 147 [range 55–700]) and Pemba Island (mean EPG 202 [range 51–803]), the *T. incognita* population in Côte d’Ivoire had low ITS1 and ITS2 α diversity relative to the *T. trichiura* populations (Figure 3; Appendix Table 4). It is unlikely that a higher allelic dropout occurred for *T. incognita* than for *T. trichiura* because the primers used in this study had 100% identity with ITS1 and ITS2 from *T. suis* reference sequences. Low genetic diversity of the *T. incognita* population might indicate that this species has recently adapted to human hosts, and its presence in nonhuman primates might be consistent with this hypothesis (*40*,*41*). Alternatively, the low diversity could be from selective pressure because of the long history of albendazole/ivermectin MDA in the region.

In conclusion, we have identified *T. incognita* in Côte d’Ivoire, which is more closely related to *T. suis* than to *T. trichiura* and is less responsive to albendazole/ivermectin combination therapy. This work demonstrates how fecal DNA metabarcoding can be used to characterize human helminth populations at the community level. The application of fecal DNA metabarcoding by using ITS1 and ITS2, as well as other markers, will be a powerful method to investigate the geographic distribution, treatment efficacy, and control strategies for *Trichuris* spp. (N. Rahman et al., unpub. data, https://doi.org/10.1101/2024.07.31.605962).

Appendix Additional information for trichuriasis in human patients from Côte d’Ivoire caused by novel species *Trichuris incognita* with low sensitivity to albendazole/ivermectin combination treatment.
