# Supplementary material for: Trichuriasis in Human Patients from Côte d’Ivoire Caused by Novel Species Trichuris incognita with Low Sensitivity to Albendazole/Ivermectin Combination Treatment
Source: Emerg Infect Dis. 2025 Jan;31(1):104–14. doi: 10.3201/eid3101.240995 (PMC11682790; doi:10.3201/eid3101.240995)
Supplement: Appendix — Additional information for trichuriasis in human patients from Côte d’Ivoire caused by novel species Trichuris incognita with low sensitivity to albendazole/ivermectin combination treatment. [file 24-0995-Techapp-s1.pdf]

Article DOI: https://doi.org/10.3201/eid3101.240995

EID cannot ensure accessibility for supplementary materials supplied by authors. Readers who have difficulty accessing supplementary content should contact the authors for assistance.

Trichuriasis in Human Patients from Côte d'Ivoire Caused by Novel Species *Trichuris incognita* with Low Sensitivity to Albendazole/Ivermectin Combination Treatment

Appendix

DNA Extraction Protocol for Fecal Samples

Washing

1) Aliquot 500 µL (250 µL, 2×) of homogenized fecal sample into the PowerBead Pro tube (from PowerFecal Pro Kit). You should first remove the beads from the bead tube and transfer them to another tube before this step. (Aliquoting the sample in 2 × 250 µL volume ensures adequate homogenization and capture of as much feces as possible in the total 500 µL of starting material).

2) To the sample (500 µL) in the PowerBead Pro tube, add 1 mL molecular grade water, briefly vortex and spin in the mini centrifuge at 13,400 rpm (≈12,000 × *g)* for 5 minutes by using an Eppendorf Mini Spin Centrifuge.

3) Discard 500 µL of supernatant and add 500 µL ddH_2O. Spin again for the same amount of time and at the same speed.

4) Discard 1,000 µL of supernatant from the previous step. Do not add more water. Spin again for 2 minutes at the same speed.

5) After spinning, carefully discard ≈250–300 µL of supernatant, depending on the nature and volume of your pellet.

6) After washing, you should now have a final volume of 200–250 μL of feces in water in your tube. Add 500 μL of CD1 solution (PowerFecal Pro Kit), transfer the beads back to this tube, vortex well and carry out the optimization steps.

Mechanical Disruption Steps, ×3

1) Snap freeze the tubes by dipping them in liquid nitrogen for ≈10 seconds or until they are completely frozen.

2) Heat at 100°C at 750 rpm for 15 minutes in a thermoblock.

3) Use the Mini Bead Beater (MBB 96) to carry out bead beating for 3 minutes. Vortex after this step.

4) Repeat steps 1 through 3 three times.

DNA Extraction using the Powerfecal Pro Kit

1) After optimization, add the remaining 300 μL of CD1 solution, bringing the total volume of CD1 solution to 800 μL. (The sample after optimization becomes very frothy, be careful while opening the caps and adding the remaining volume of CD1)

2) Secure the PowerBead Pro Tube horizontally on a Vortex Adaptor and vortex at maximum speed for 10 minutes.

3) Centrifuge the PowerBead Pro Tube at 15,000 × *g* for **2 minutes** (centrifugation time changed compared with actual protocol).

4) Transfer the supernatant to a clean 2 mL microcentrifuge tube (provided). Transfer **≈800–850 μL of supernatant** (depending on the nature and volume of the pellet).

5) Add 200 μL of Solution CD2 and vortex for 5 seconds.

6) Centrifuge at 15,000 × *g* for 1 minute. Avoiding the pellet, transfer up to **800 μL of supernatant** (depending on the volume of your pellet) to a clean 2 mL microcentrifuge tube (provided).

7) Add 600 μL of Solution CD3 and vortex for 5 seconds.

8) Load 650 μL of the lysate onto an MB Spin Column and centrifuge at 15,000 × *g* for 1 minute.

9) Discard the flow-through and repeat step 8 to ensure that all of the lysate has passed through the MB Spin Column.

10) Carefully place the MB Spin Column into a clean 2 ml collection tube (provided). Avoid splashing any flow-through onto the MB Spin Column.

11) Add 500 µL of Solution EA to the MB Spin Column. Centrifuge at 15,000 × *g* for 1 min.

12) Discard the flow-through and place the MB Spin Column back into the same 2 mL Collection Tube.

13) Add 500 µL of Solution C5 to the MB Spin Column. Centrifuge at 15,000 × *g* for 1 minute.

14) Discard the flow-through and place the MB Spin Column into a new 2 mL collection tube (provided).

15) Centrifuge at up to 15,000 × *g* for 2 minutes. Carefully place the MB Spin Column into a new 1.5 mL elution tube (provided).

16) Add 100 µL of Solution C6 to the center of the white filter membrane. **Incubate at room temperature for 1 minute.**

17) Centrifuge at 15,000 × *g* for 1 minute. Discard the MB Spin Column. Store the DNA at −20°C.

References

1. Ravasi DF, O'Riain MJ, Davids F, Illing N. Phylogenetic evidence that two distinct *Trichuris* genotypes infect both humans and non-human primates. PLoS One. 2012;7:e44187. PubMed https://doi.org/10.1371/journal.pone.0044187

2. Ghai RR, Simons ND, Chapman CA, Omeja PA, Davies TJ, Ting N, et al. Hidden population structure and cross-species transmission of whipworms (*Trichuris* sp.) in humans and non-human primates in Uganda. PLoS Negl Trop Dis. 2014;8:e3256. PubMed https://doi.org/10.1371/journal.pntd.0003256

3. Nissen S, Al-Jubury A, Hansen TVA, Olsen A, Christensen H, Thamsborg SM, et al. Genetic analysis of *Trichuris suis* and *Trichuris trichiura* recovered from humans and pigs in a sympatric setting in Uganda. Vet Parasitol. 2012;188:68–77. PubMed https://doi.org/10.1016/j.vetpar.2012.03.004

4. Liu GH, Zhou W, Nisbet AJ, Xu MJ, Zhou DH, Zhao GH, et al. Characterization of *Trichuris trichiura* from humans and *T. suis* from pigs in China using internal transcribed spacers of nuclear ribosomal DNA. J Helminthol. 2014;88:64–8. PubMed https://doi.org/10.1017/S0022149X12000740

5. Cavallero S, De Liberato C, Friedrich KG, Di Cave D, Masella V, D'Amelio S, et al. Genetic heterogeneity and phylogeny of *Trichuris* spp. from captive non-human primates based on ribosomal DNA sequence data. Infect Genet Evol. 2015;34:450–6. PubMed https://doi.org/10.1016/j.meegid.2015.06.009

6. Xie Y, Zhao B, Hoberg EP, Li M, Zhou X, Gu X, et al. Genetic characterisation and phylogenetic status of whipworms (*Trichuris* spp.) from captive non-human primates in China, determined by nuclear and mitochondrial sequencing. Parasit Vectors. 2018;11:516. PubMed https://doi.org/10.1186/s13071-018-3100-5

7. Cutillas C, de Rojas M, Ariza C, Ubeda JM, Guevara D. Molecular identification of *Trichuris vulpis* and *Trichuris suis* isolated from different hosts. Parasitol Res. 2007;100:383–9. PubMed https://doi.org/10.1007/s00436-006-0275-z

8. Hawash MBF, Andersen LO, Gasser RB, Stensvold CR, Nejsum P. Mitochondrial genome analyses suggest multiple *Trichuris* species in humans, baboons, and pigs from different geographical regions. PLoS Negl Trop Dis. 2015;9:e0004059. PubMed https://doi.org/10.1371/journal.pntd.0004059

9. Liu GH, Gasser RB, Su A, Nejsum P, Peng L, Lin RQ, et al. Clear genetic distinctiveness between human- and pig-derived *Trichuris* based on analyses of mitochondrial datasets. PLoS Negl Trop Dis. 2012;6:e1539. PubMed https://doi.org/10.1371/journal.pntd.0001539

10. Søe MJ, Nejsum P, Seersholm FV, Fredensborg BL, Habraken R, Haase K, et al. Ancient DNA from latrines in northern Europe and the Middle East (500 BC–1700 AD) reveals past parasites and diet. PLoS One. 2018;13:e0195481. PubMed https://doi.org/10.1371/journal.pone.0195481

11. Doyle SR, Søe MJ, Nejsum P, Betson M, Cooper PJ, Peng L, et al. Population genomics of ancient and modern *Trichuris trichiura*. Nat Commun. 2022;13:3888. PubMed https://doi.org/10.1038/s41467-022-31487-x

12. Mohandas N, Pozio E, La Rosa G, Korhonen PK, Young ND, Koehler AV, et al. Mitochondrial genomes of *Trichinella* species and genotypes—a basis for diagnosis, and systematic and epidemiological explorations. Int J Parasitol. 2014;44:1073–80. PubMed https://doi.org/10.1016/j.ijpara.2014.08.010

13. Lavrov DV, Brown WM. *Trichinella spiralis* mtDNA: a nematode mitochondrial genome that encodes a putative ATP8 and normally structured tRNAS and has a gene arrangement relatable to those of coelomate metazoans. Genetics. 2001;157:621–37. PubMed https://doi.org/10.1093/genetics/157.2.621

14. Petružela J, Ribas A, de Bellocq JG. Mitogenomics and evolutionary history of rodent whipworms (*Trichuris* spp.) originating from three biogeographic regions. Life (Basel). 2021;11:540. PubMed https://doi.org/10.3390/life11060540

15. Wang HB, Zhang HJ, Song LL, Zhu L, Chen M, Ren GJ, et al. Morphological and molecular confirmation of the validity of *Trichuris rhinopiptheroxella* in the endangered golden snub-nosed monkey (*Rhinopithecus roxellana*). J Helminthol. 2019;93:601–7. PubMed https://doi.org/10.1017/S0022149X18000500

16. Ahmad AA, Shabbir MAB, Xin Y, Ikram M, Hafeez MA, Wang C, et al. Characterization of the complete mitochondrial genome of a whipworm *Trichuris skrjabini* (Nematoda: Trichuridae). Genes (Basel). 2019;10:438. PubMed https://doi.org/10.3390/genes10060438

17. Liu GH, Gasser RB, Nejsum P, Wang Y, Chen Q, Song HQ, et al. Mitochondrial and nuclear ribosomal DNA evidence supports the existence of a new *Trichuris* species in the endangered françois' leaf-monkey. PLoS One. 2013;8:e66249. PubMed https://doi.org/10.1371/journal.pone.0066249

18. Rivero J, Cutillas C, Callejon R. *Trichuris trichiura* (Linnaeus, 1771) from human and non-human primates: morphology, biometry, host specificity, molecular characterization, and phylogeny. Front. Vet. Sci. 2021;7:626120. PubMed https://doi.org/10.3389/fvets.2020.626120

Appendix Table 1. Mean fecal egg count of patient samples from Côte d'Ivoire, Lao PDR, and Pemba island, Tanzania*

Patient population	Patient ID no.	Mean EPG
Côte d'Ivoire	BL-A4463	835
Côte d'Ivoire	BL-A4572	201
Côte d'Ivoire	BL-A4574	193.5
Côte d'Ivoire	BL-A4617	255.5
Côte d'Ivoire	BL-A4651	238
Côte d'Ivoire	BL-A4654	1151.5
Côte d'Ivoire	BL-B0133	132.5
Côte d'Ivoire	BL-B0393	189.5
Côte d'Ivoire	BL-B0704	230
Côte d'Ivoire	BL-B1977	237
Côte d'Ivoire	BL-B2032	152.5
Côte d'Ivoire	BL-B6033	353.5
Côte d'Ivoire	BL-B7235	163
Côte d'Ivoire	BL-B7534	262
Côte d'Ivoire	BL-B8460	384.5
Côte d'Ivoire	BL-B8563	354.5
Côte d'Ivoire	BL-B8744	110.5
Côte d'Ivoire	BL-B8745	730.5
Côte d'Ivoire	BL-B8746	383.5
Côte d'Ivoire	BL-B8823	555.5
Côte d'Ivoire	BL-B8835	381
Côte d'Ivoire	BL-B9113	318.5
Côte d'Ivoire	BL-B9674	243
Lao PDR	BL-9625	699.5
Lao PDR	BL-2445	506
Lao PDR	BL-7181	253.5
Lao PDR	BL-7180	261
Lao PDR	BL-A1221	242.5
Lao PDR	BL-A1244	224
Lao PDR	BL-7374	148
Lao PDR	BL-A0421	186.5
Lao PDR	BL-9754	123.5
Lao PDR	BL-7208	192.5
Lao PDR	BL-9624	139
Lao PDR	BL-9753	128
Lao PDR	BL-A1303	145.5
Lao PDR	BL-7192	96.5
Lao PDR	BL-A0988	130
Lao PDR	BL-A0986	159
Lao PDR	BL-1316	90.5
Lao PDR	BL-A2774	95
Lao PDR	BL-A0293	94
Lao PDR	BL-9023	84.5
Lao PDR	BL-A0944	84.5
Lao PDR	BL-A0992	97.5
Lao PDR	BL-7480	105
Lao PDR	BL-A1581	73
Lao PDR	BL-A1621	72.5
Lao PDR	BL-A0884	72.5
Lao PDR	BL-A3012	103.5
Lao PDR	BL-A1391	97
Lao PDR	BL-7528	68
Lao PDR	BL-A1834	84.5
Lao PDR	BL-A2775	75
Lao PDR	BL-9622	91
Lao PDR	BL-A0985	71.5
Lao PDR	BL-A1261	100
Lao PDR	BL-7065	97
Lao PDR	BL-7415	54.5
Lao PDR	BL-7008	88.5
Pemba	BL-43	55.5
Pemba	BL-52	133.5
Pemba	BL-55	70
Pemba	BL-56	170.5
Pemba	BL-58	79
Pemba	BL-59	68.5
Pemba	BL-90	85
Pemba	BL-103	400

Patient population	Patient ID no.	Mean EPG
Pemba	BL-104	80.5
Pemba	BL-117	51
Pemba	BL-144	72
Pemba	BL-152	262.5
Pemba	BL-314	214.5
Pemba	BL-724	77.5
Pemba	BL-1095	76
Pemba	BL-1108	87.5
Pemba	BL-1366	55.5
Pemba	BL-1465	214
Pemba	BL-1516	92
Pemba	BL-2155	162.5
Pemba	BL-2163	429
Pemba	BL-2165	309.5
Pemba	BL-2175	150
Pemba	BL-2245	152
Pemba	BL-3015	92
Pemba	BL-3444	76
Pemba	BL-4176	492.5
Pemba	BL-713	802.5
Pemba	BL-4336	243.5
Pemba	BL-396	280
Pemba	BL-3017	724.5

*Fecal egg counts were calculated by using quadruplicate Kato-Katz analyses for each sample. EPG, eggs per gram; ID, identification, Lao PDR, Lao People's Democratic Republic.

Appendix Table 2. Primers used for fecal DNA metabarcoding of *Trichuris* spp. loci

Primer name	Primer sequence
Trichuris_ITS1_F	5′-ACTGGCCGAACCAAGCCATC-3′
Trichuris_ITS1_R	5′-TCTACGAGCCAAGTGATCCAC-3′
Trichuris_ITS2_F	5′-ATGTCGACGCTACGCCTGTC-3′
Trichuris_ITS2_R	5′-TAGCCTCGTCTGATCTGAGG-3′
Trichuris_COX1_F	5′-TGGCATAGATATTGACACTCG-3′
Trichuris_COX1_R	5′-AAGCAGTCGTTGTAGTCTAAG-3′
Trichuris_NAD1_F	5′-ATACTTCAACCCGTCCTAGATG-3′
Trichuris_NAD1_R	5′-TCGAGTAAATCAAATGGAGTCC-3′
Trichuris_NAD4_F	5′-AGAGTCGGGATGTACTAGCGTA-3′
Trichuris_NAD4_R	5′-AGTCGGACCATTTACTTGAGTTC-3′

Appendix Table 3. *Trichuris* spp. sequences from GenBank used for phylogenetic analyses of ribosomal and mitochondrial loci*

GenBank accession no. or nomenclature	Host	Location	Marker	Reference
GQ301555	Human	Cameroon	ITS1–5.8S–ITS-2	*(1)*
KJ588159	Human	Uganda	ITS-2	*(2)*
JN181817	Human	Jamaica	ITS-2	*(3)*
JN181820	Human	Jamaica	ITS-2	*(3)*
JN181850	Human	Uganda	ITS-2	*(3)*
AM992984	Human	China	ITS-1–5.8S–ITS-2	*(4)*
AM992997	Human	China	ITS-1–5.8S–ITS-2	*(4)*
KJ588071	Human	Uganda	ITS-1	*(2)*
KP336484	Vervet monkey	Italy	ITS1–5.8S–ITS-2	*(5)*
JF690945	Lion-tailed macaque	Czech Republic	ITS-2	Unpublished
KJ588162	Red colobus	Uganda	ITS-2	*(2)*
KT344830	White-cheeked gibbon	China	ITS-1–5.8S–ITS-2	*(6)*
MH390366	White-cheeked gibbon	China	ITS-1–5.8S–ITS-2	*(6)*
AM992999	Pig	China	ITS-1–5.8S–ITS-2	*(4)*
MG656442	Pig	China	ITS-1	Unpublished
MG656443	Pig	China	ITS-2	Unpublished
AJ249966	Pig	Spain	ITS-2	*(7)*
AP017704	Human	Japan	Mitochondrion	Unpublished
KT449826	Human	Uganda	Mitochondrion	*(8)*

GenBank accession no. or nomenclature	Host	Location	Marker	Reference
KT449824	Hamadryas baboon	Denmark	Mitochondrion	*(8)*
KT449825	Olive baboon	USA	Mitochondrion	*(8)*
KT449823	Pig	Denmark	Mitochondrion	*(8)*
GU070737	Pig	China	Mitochondrion	*(9)*
KY368772	Human	Denmark	Mitochondrion	*(10)*
MN_ESP_MAL_CG_001	*Colobus* monkey	Spain	Mitochondrion	*(11)*
MN_ESP_MAL_CG_002	*Colobus* monkey	Spain	Mitochondrion	*(11)*
MN_CHN_GUA_LM_008	Francois leaf monkey	China	Mitochondrion	*(11)*
MN_UGA_KAB_HS_005	Human	Uganda	Mitochondrion	*(11)*
MN_DNK_COZ_PH_001	Baboon	Denmark	Mitochondrion	*(11)*
MN_HND_SAL_HS_001	Human	Honduras	Mitochondrion	*(11)*
MN_HND_OLA_HS_001	Human	Honduras	Mitochondrion	*(11)*
MN_CHN_GUA_HS_001	Human	China	Mitochondrion	*(11)*
MN_CHN_GUA_HS_005	Human	China	Mitochondrion	*(11)*
NC_025751	Coyote	USA	Mitochondrion	*(12)*
NC_025754	Human	Thailand	Mitochondrion	*(12)*
NC_025749	Raccoon	Russia	Mitochondrion	*(12)*
NC_002681	NA, larvae	USA	Mitochondrion	*(13)*
MZ229684	Vole	Czech Republic	Mitochondrion	*(14)*
JQ996231	Yak	China	Mitochondrion	*(9)*
MZ229690	Natal multimammate mouse	Tanzania	Mitochondrion	*(14)*
KY368775	N/A - Latrine	Denmark	Mitochondrion	*(10)*
JQ996232	Addax	China	Mitochondrion	*(9)*
MG189593	Golden snub-nosed monkey	China	Mitochondrion	*(15)*
MK333462	Sheep	China	Mitochondrion	*(16)*
MZ229685	Greater bandicoot rat	Laos	Mitochondrion	*(14)*
MZ229688	Mahomet mouse	Ethiopia	Mitochondrion	*(14)*
MZ229686	Guinea multimammate mouse	Ethiopia	Mitochondrion	*(14)*
KC461179	Francois leaf monkey	China	Mitochondrion	*(17)*
MZ229689	Misonne's soft-furred mouse	Kenya	Mitochondrion	*(14)*
MW448471	Barbary macaque	Spain	Mitochondrion	*(18)*
KT449822	Pig	Denmark	Mitochondrion	*(8)*
GU385218	Human	China	Mitochondrion	*(9)*
KY368765	NA, latrine	Netherlands	Mitochondrion	*(10)*
KY368773	NA, latrine	Denmark	Mitochondrion	*(10)*
MW448470	Barbary macaque	Spain	Mitochondrion	*(18)*
MW448472	Guinea baboon	Spain	Mitochondrion	*(18)*

*ITS-1, internal transcribed spacer 1; ITS-2, internal transcribed spacer 2; NA, not applicable.

Appendix Table 4. Genetic diversity of the rDNA loci

	ITS-1				ITS-2			
Region	No. samples*	No. ASVs	H_d	π	No. samples*	No. ASVs	H_d	π
Côte d'Ivoire	21	7	0.52	0.001	15	3	0.56	0.000
Laos	34	92	0.98	0.003	26	32	0.82	0.006
Pemba Island	29	63	0.96	0.003	23	13	0.69	0.006

*Number of samples sequenced. ASV, amplicon sequence variant; Hd, ASV heterozygosity; ITS-1, internal transcribed spacer 1; ITS-2, internal transcribed spacer 2; π, nucleotide diversity.

Appendix Table 5. Average pairwise nucleotide identity between *Trichuris* sp. from *Colobus* monkeys (n = 2), *T. suis* (n = 3), and *T. incognita* (n = 8) clades

	Nucleotide identity for *cox-1*, %			Nucleotide identity for all protein coding genes, %		
Species	*Trichuris* sp. *Colobus* monkey	*T. suis*	*T. incognita*	*Trichuris* sp., *Colobus* monkey	*T. suis*	*T. incognita*
Trichuris sp., *Colobus* monkey	99.6*	77.8	80.2	98.1*	71.6	73.1
T. suis	77.8	93.1*	78.9	71.6	91.7	71.7
T. incognita	80.2	78.9	99.4*	73.1	71.7	94*

*Indicates average pairwise nucleotide identity between samples of the same species/clade.

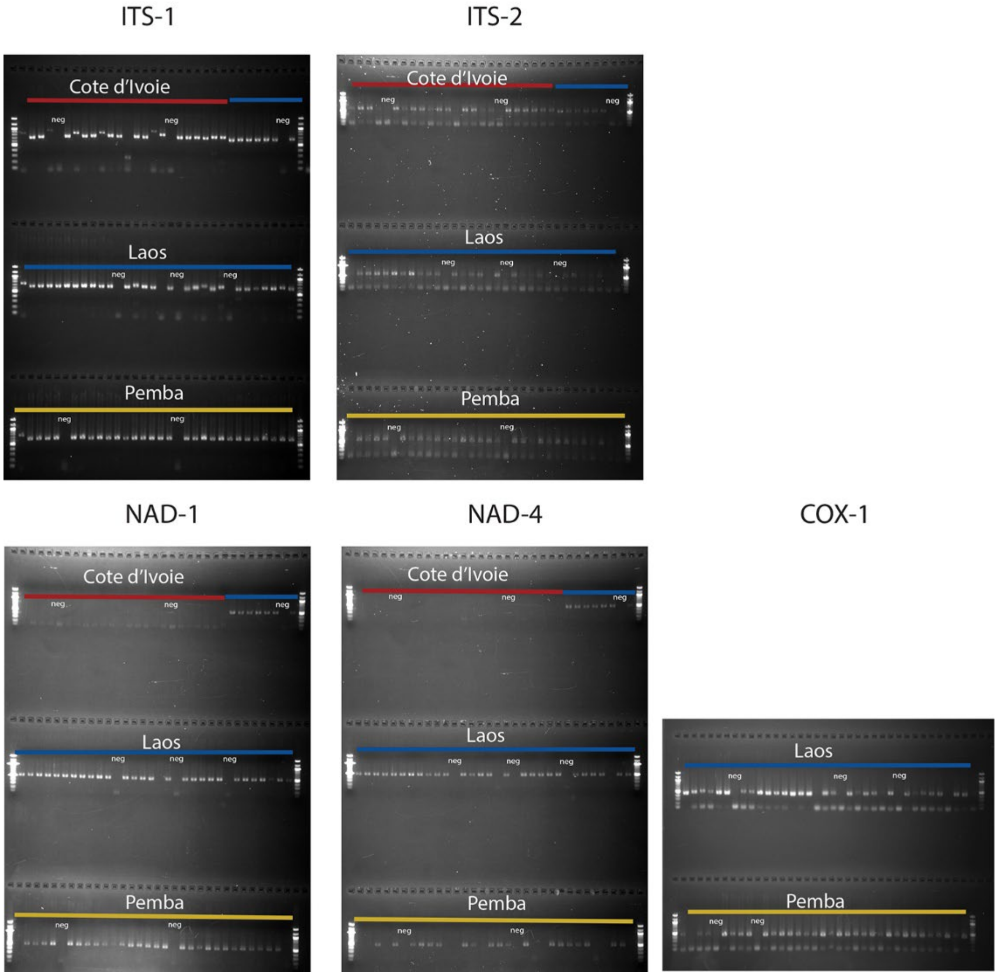

Appendix Figure 1. Gel electrophoresis results from PCR amplicon generation. Gel electrophoresis of PCR-amplified ITS-1, ITS-2 and mitochondrial genes *cox-1, nad-1,* and *nad-4* from patient fecal samples collected from Côte d'Ivoire, Laos, and Pemba Island, Tanzania. Species-specific primers designed by using *T. trichiura* reference sequences (Genbank accession nos. NC_017750, GU385218, AP017704, and KT449825) successfully generated 32 *cox-1*, 35 *nad-1*, and 36 *nad-4* amplicons from samples collected in Laos and 29 *cox-1*, 28 *nad-1*, and 19 *nad-4* amplicons from samples collected in Pemba Island. No amplicons were generated from any of the 22 samples collected in Côte d'Ivoire. ITS-1, internal transcribed spacer 1; ITS-2, internal transcribed spacer 2.

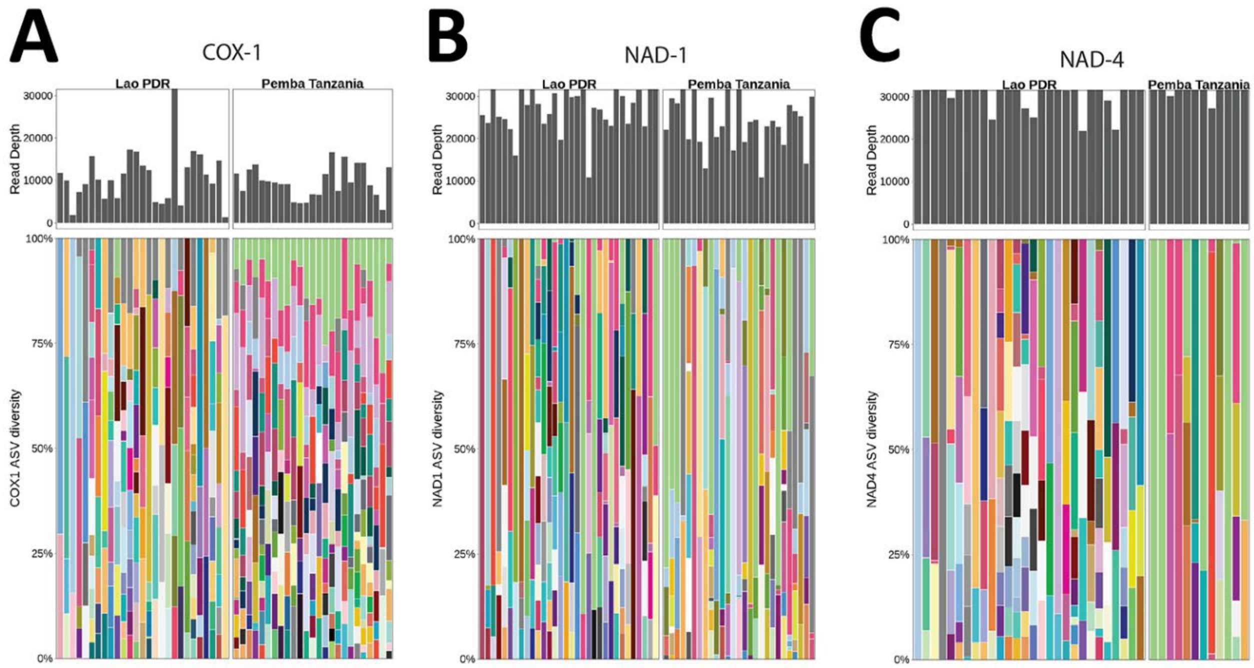

Appendix Figure 2. Read depth and frequency of *Trichuris* spp. ASVs of *cox-1, nad-1,* and *nad-4* genes from patient fecal samples collected from Laos, and Pemba Island, Tanzania. A–C) ASVs were identified by amplicon sequencing of *cox-1* (A), *nad-1* (B), and *nad-4* (C) gene markers. Histograms indicate read depths of each gene sequence. Bar graphs indicate relative frequencies of different ASVs of *Trichuris* mitochondrial markers amplified from each region. Lower bar plots represent the relative abundance of the ASVs present in each sample from Laos and Pemba . Metabarcoding data were generated from 27 (Laos) and 25 (Pemba) samples for the 430-bp *cox-1* amplicon, from 32 (Laos) and 27(Pemba) samples for the 470-bp *nad-1* amplicon, and from 28 (Laos) and 12 (Pemba) samples for the 446-bp *nad-4* amplicon. Paired-end reads from each of the 3 gene markers were merged and concatenated to produce average mapped read depths of 10,300 (range 1,355–32,600) reads for *cox-1*, 26,490 (range 10,791–56,330) reads for *nad-1*, and 36,350 (range 21,936–55,346) reads for *nad-4*.

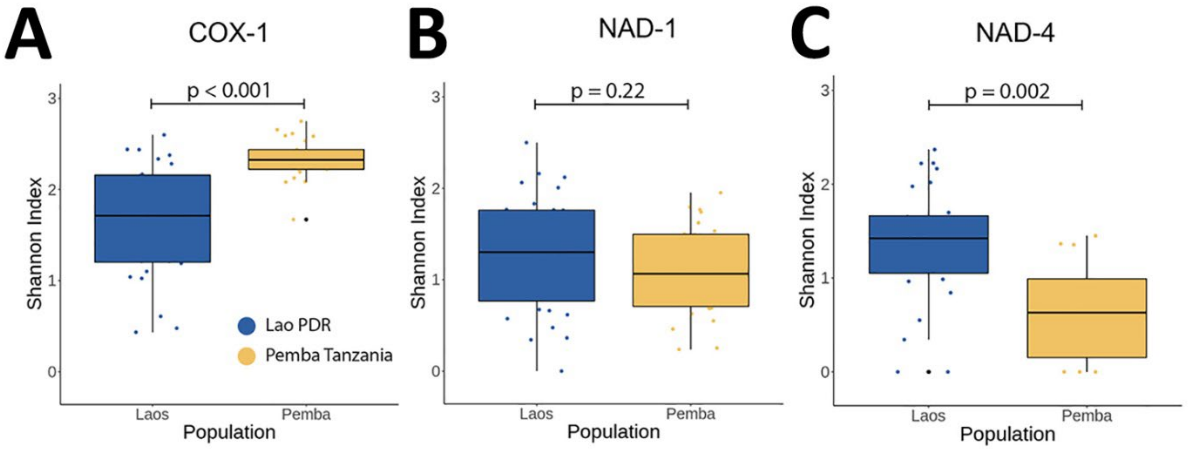

D

Region	cox-1				nad-1				nad-4			
	N	N_{ASV}	H_d	π	N	N_{ASV}	H_d	π	N	N_{ASV}	H_d	π
Laos	33	166	0.98	0.038	31	121	0.98	0.012	28	147	0.98	0.014
Pemba island	29	129	0.96	0.022	27	106	0.93	0.034	13	27	0.80	0.008

Appendix Figure 3. Alpha diversity at the mitochondrial DNA loci. A–C) Shannon-Wiener Index values, were determined for *Trichuris* spp. *cox-1* (A), *nad-1* (B), and *nad-4* (C) amplicon sequence variants generated by sequencing fecal sample DNA from patients in Laos, and Pemba Island, Tanzania. Horizontal lines within boxes indicate medians; box tops and bottoms indicate upper (third) and lower (first) quartiles; error bars (whiskers) indicate minimum and maximum values. Pairwise *t*-tests were used to calculate p values. D) Genetic diversity observed at the 3 mitochondrial loci. ASV, amplicon sequence variant; N, number of samples; N_{ASV}, number of ASVs; H_d, ASV heterozygosity; π, nucleotide diversity.

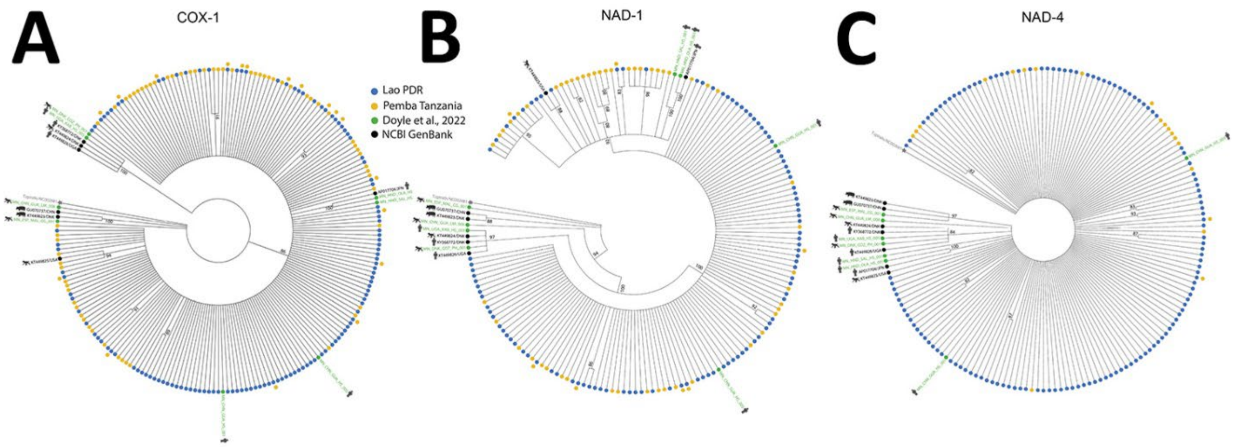

Appendix Figure 4. Maximum-likelihood phylogenetic trees of mitochondrial *cox-1, nad-1,* and *nad-4* amplicon sequence variants (ASVs) from patient fecal samples collected in Laos and Pemba Island, Tanzania. Trees indicate *cox-1* (A), *nad-1* (B), and *nad-4* (C) ASVs amplified from fecal samples from patients in Laos and Pemba Island as well as additional *Trichuris* reference sequences from pigs, humans, and nonhuman primates in GenBank. Gamma-distributed rate variation, general time reversible model was chosen for the mitochondrial markers. *Trichinella spiralis* (GenBank accession: no. NC002681) was used as the outgroup. Each tip of the tree is an ASV or a sequence from GenBank, and the color represents the geographic region in which the ASV was found. Trees not to scale.

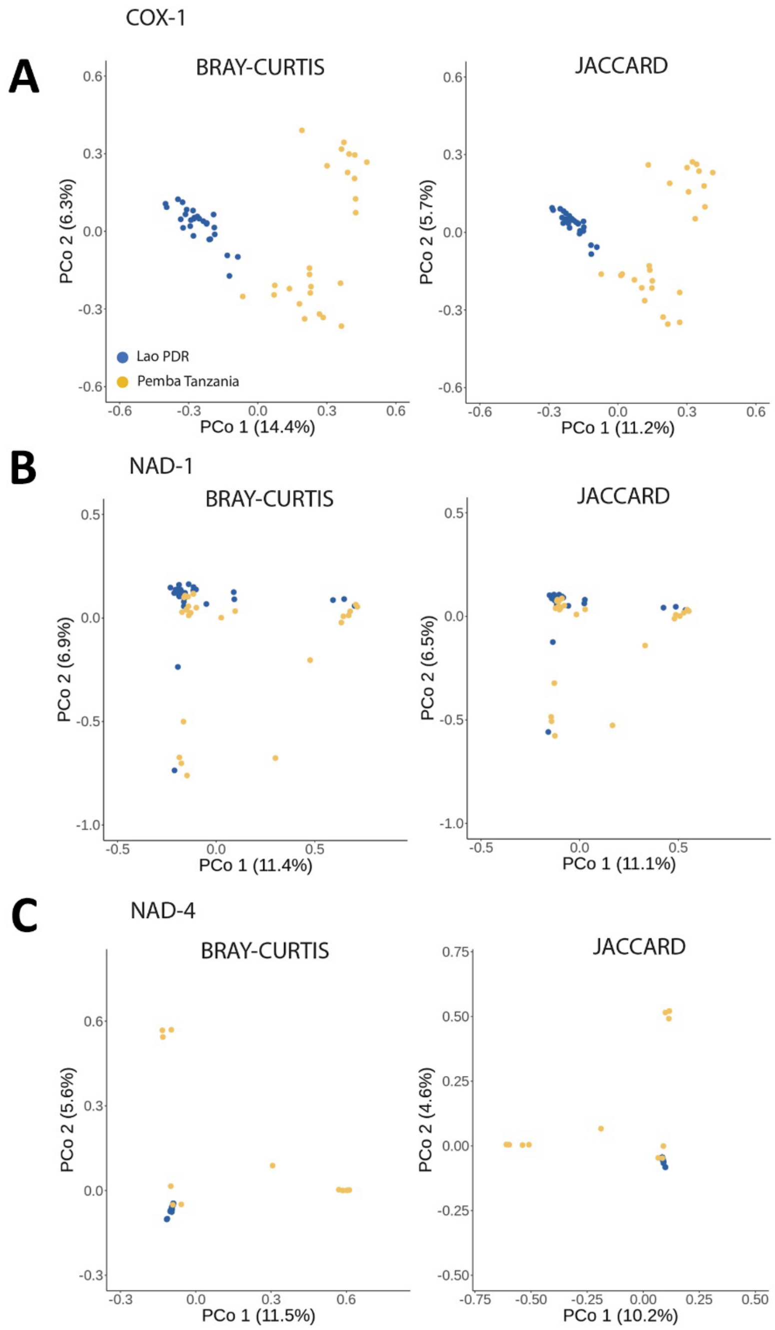

Appendix Figure 5. Multidimensional metric analysis indicating beta diversity of *cox-1, nad-1,* and *nad-4* amplicon sequence variant (ASV) data generated by amplicon sequencing of patient fecal DNA samples from Laos and Pemba Island, Tanzania. A–C) Principal coordinate analysis plots of mitochondrial *cox-1* (A)*, nad-1* (B)*,* and *nad-4* (C) loci from samples collected from Laos and Pemba Island. Multidimensional metric analysis was performed by using the Bray-Curtis dissimilarity matrix, which calculates distances according to the abundance of ASVs, and the Jaccard index, which calculates distances according to the presence or absence of ASVs. Each point is an individual patient sample, and the points are colored according to the geographic region in which the samples were collected. PCo, principal coordinates.

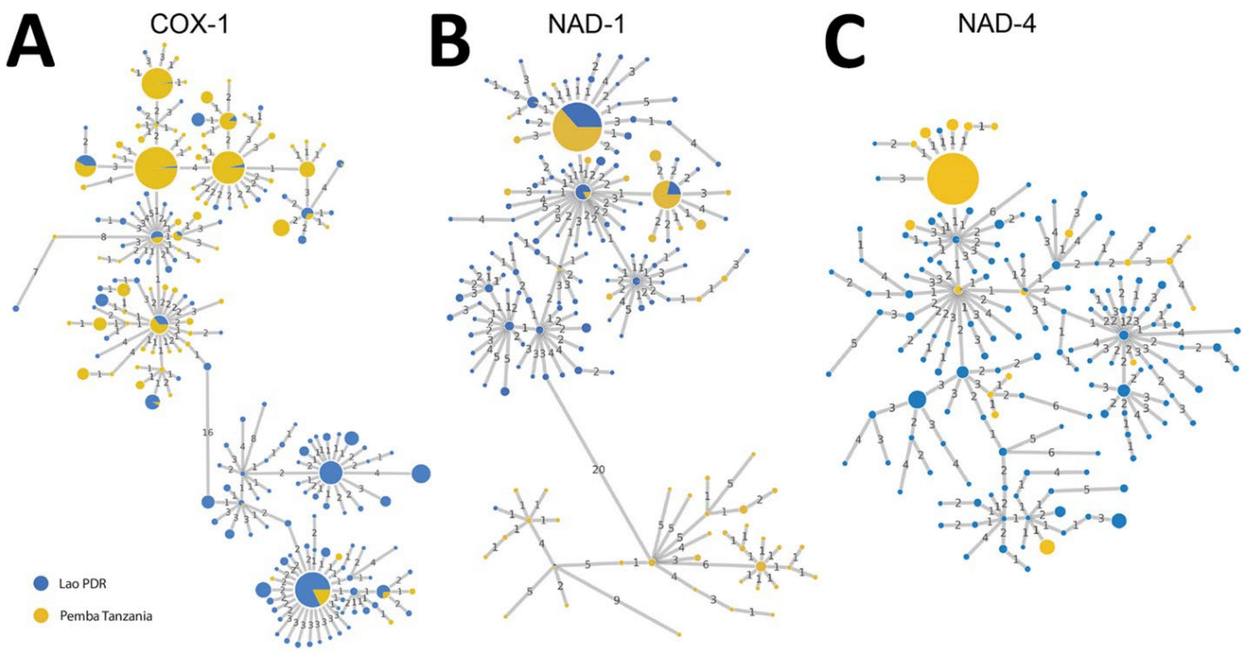

Appendix Figure 6. Haplotype network of *Trichuris* amplicon sequence variants (ASVs) of mitochondrial markers generated by amplicon sequencing of patient fecal samples from Laos and Pemba Island, Tanzania. A–C) Statistical parsimony haplotype networks are indicated for mitochondrial *cox-1* (A), *nad-1* (B), and *nad-4* (C) ASVs. Colored circles indicate the region, and size of each circle indicates the ASV frequency. Numbers on connecting lines indicate the number of nucleotide differences between adjacent haplotypes. Haplotype networks show the major high frequency ASVs differed between the 2 regions for all 3 markers and most low-frequency ASVs were unique to either region.

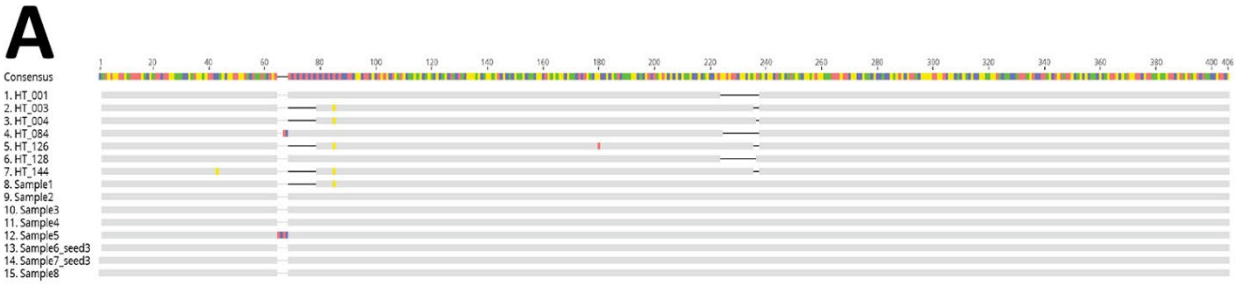

B

	HT_001	HT_003	HT_004	HT_084	HT_126	HT_128	HT_144	Sample1	Sample2	Sample3	Sample4	Sample5	Sample6	Sample7	Sample8
HT_001		94.2	94.0	99.2	94.0	99.7	94.0	93.8	96.5	96.5	96.5	95.6	96.5	96.5	96.5
HT_003	94.2		99.7	94.0	99.7	94.0	99.7	99.5	96.8	96.8	96.8	95.8	96.8	96.8	96.8
HT_004	94.0	99.7		93.8	99.5	93.8	99.5	99.7	97.0	97.0	97.0	96.0	97.0	97.0	97.0
HT_084	99.2	94.0	93.8		93.8	99.0	93.8	93.5	96.3	96.3	96.3	96.3	96.3	96.3	96.3
HT_126	94.0	99.7	99.5	93.8		93.8	99.5	99.2	96.5	96.5	96.5	95.6	96.5	96.5	96.5
HT_128	99.7	94.0	93.8	99.0	93.8		93.8	94.0	96.8	96.8	96.8	95.8	96.8	96.8	96.8
HT_144	94.0	99.7	99.5	93.8	99.5	93.8		99.2	96.5	96.5	96.5	95.6	96.5	96.5	96.5
Sample1	93.8	99.5	99.7	93.5	99.2	94.0	99.2		97.3	97.3	97.3	96.3	97.3	97.3	97.3
Sample2	96.5	96.8	97.0	96.3	96.5	96.8	96.5	97.3		100.0	100.0	99.0	100.0	100.0	100.0
Sample3	96.5	96.8	97.0	96.3	96.5	96.8	96.5	97.3	100.0		100.0	99.0	100.0	100.0	100.0
Sample4	96.5	96.8	97.0	96.3	96.5	96.8	96.5	97.3	100.0	100.0		99.0	100.0	100.0	100.0
Sample5	95.6	95.8	96.0	96.3	95.6	95.8	95.6	96.3	99.0	99.0	99.0		99.0	99.0	99.0
Sample6	96.5	96.8	97.0	96.3	96.5	96.8	96.5	97.3	100.0	100.0	100.0	99.0		100.0	100.0
Sample7	96.5	96.8	97.0	96.3	96.5	96.8	96.5	97.3	100.0	100.0	100.0	99.0	100.0		100.0
Sample8	96.5	96.8	97.0	96.3	96.5	96.8	96.5	97.3	100.0	100.0	100.0	99.0	100.0	100.0	

Appendix Figure 7. Sequence alignments of ITS-1 amplicon sequence variants from patient fecal samples collected in Côte d’Ivoire with whole-genome sequences from 8 adult worms obtained through an expulsion study. A) Multiple sequence alignments of the 406-bp ribosomal internal transcribed spacer (ITS) 1 sequences generated from the *Trichuris* population in Côte d’Ivoire by using amplicon sequencing of fecal samples and ITS-1 sequences extracted after whole-genome sequencing of adult worms. Dissimilarities are highlighted by black lines or different colors. B) Distance matrix displaying the percentage identity among the ITS-1 sequences.

A

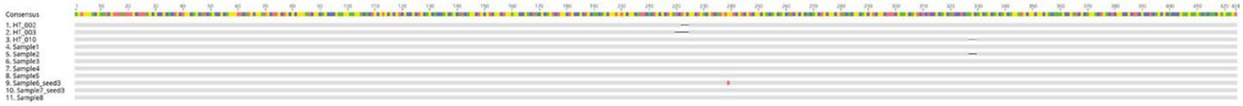

B

	HT_002	HT_003	HT_010	Sample1	Sample2	Sample3	Sample4	Sample5	Sample6	Sample7	Sample8
HT_002		99.5	98.6	99.3	98.6	99.3	99.3	99.3	99.1	99.3	99.3
HT_003	99.5		98.1	98.8	98.1	98.8	98.8	98.8	98.6	98.8	98.8
HT_010	98.6	98.1		99.3	100.0	99.3	99.3	99.3	99.1	99.3	99.3
Sample1	99.3	98.8	99.3		99.3	100.0	100.0	100.0	99.8	100.0	100.0
Sample2	98.6	98.1	100.0	99.3		99.3	99.3	99.3	99.1	99.3	99.3
Sample3	99.3	98.8	99.3	100.0	99.3		100.0	100.0	99.8	100.0	100.0
Sample4	99.3	98.8	99.3	100.0	99.3	100.0		100.0	99.8	100.0	100.0
Sample5	99.3	98.8	99.3	100.0	99.3	100.0	100.0		99.8	100.0	100.0
Sample6	99.1	98.6	99.1	99.8	99.1	99.8	99.8	99.8		99.8	99.8
Sample7	99.3	98.8	99.3	100.0	99.3	100.0	100.0	100.0	99.8		100.0
Sample8	99.3	98.8	99.3	100.0	99.3	100.0	100.0	100.0	99.8	100.0	

Appendix Figure 8. Sequence alignments of ITS-2 amplicon sequence variants from patient fecal samples collected in Côte d’Ivoire with whole-genome sequences from 8 adult worms obtained through an expulsion study. A) Multiple sequence alignments of the 424-bp ribosomal internal transcribed spacer (ITS) 2 sequences generated from the *Trichuris* population in Côte d’Ivoire by using amplicon sequencing of fecal samples and ITS-2 sequences extracted after whole-genome sequencing of adult worms. Dissimilarities are highlighted by black lines or different colors. B) Distance matrix displaying the percentage identity among the ITS-2 sequences.
